# Oral microbiota signatures in obesity with or without acanthosis nigricans in a Chinese cohort

**DOI:** 10.1099/jmm.0.002020

**Published:** 2025-06-04

**Authors:** Yujing Tang, Qin Li, Zhengyun Ren, Nianwei Wu, Hongmei Zhu, Tongtong Zhang, Wei Yi, Wantao Ju, Yanjun Liu, Junqing Hu

**Affiliations:** 1 Obesity and Metabolism Medicine-Engineering Integration Laboratory, Department of General Surgery, The Third People’s Hospital of Chengdu, Chengdu, 610031, PR China; 2 Center of Gastrointestinal and Minimally Invasive Surgery, Department of General Surgery, The Third People’s Hospital of Chengdu, Chengdu, 610031, PR China; 3 The Center for Obesity and Metabolic Health, Department of General Surgery, The Third People’s Hospital of Chengdu, Chengdu, 610031, PR China; 4 Medical Research Center, The Third People’s Hospital of Chengdu, Chengdu, 610031, PR China; 5 School of Life Science and Engineering, Southwest Jiaotong University, Chengdu, 610031, PR China

**Keywords:** acanthosis nigricans, bacteria, obesity, oral microbiota, *Prevotella*

## Abstract

**Introduction.** The oral microbiota is the second most complex microbial community in the human body. It has been suggested that poor oral health may be associated with an increased risk of obesity.

**Hypothesis/Gap Statement.** However, both previous observational and mechanistic studies on oral microbiota do not take into account the obesity-related acanthosis nigricans (AN), which is the most common dermatological manifestation in individuals with obesity.

**Aim.** This study aimed to investigate the altered composition, function and diagnostic value of the oral microbiota in obesity with or without acanthosis nigricans (AN).

**Methodology.** We characterized the oral bacteria signature in a Chinese cohort (ChiCTR2300073353) of 99 patients with obesity and obesity-related AN (Ob_AN) and 50 healthy controls using 16S rRNA gene V3–V4 region sequencing.

**Results.** The microbial richness (abundance-based coverage estimators and observed species indices) was significantly greater in the Ob_AN and obesity groups than in the control group; however, microbial diversity (Shannon index) did not differ significantly. Distinct separation in the microbial community amongst the three groups was observed. *Prevotella* species, including *Prevotella melaninogenica*, *Prevotella nanceiensis* and *Prevotella pallens*, were associated with composition alterations and predicted functions (significant downregulation of ATP-binding cassette transporters) associated with microbial dysbiosis in the obesity and Ob_AN groups. Moreover, *Prevotella* and *Lautropia* genera assessments could indicate obesity and obesity-related AN risk.

**Conclusions.** The notable reduction of plenty of oral microbiota and high levels of *Prevotella* spp. may play a critical role in obesity with AN. Oral microbiota may serve as biomarkers for diagnosing, preventing and even treating obesity-related AN.

**Impact Statement**

The oral microbiota and its potential functions are significantly altered in people with obesity and AN. High levels of *Prevotella* spp., especially *Prevotella melaninogenica*, may contribute to the biomarkers of obesity-related AN. Oral microbiota assessments are potentially useful for identifying obesity and obesity-related AN risk. Our findings that oral microbiota imbalance and dysfunction may be associated with obesity-related AN provide a foundation for further investigations. Our results highlight the importance of considering oral *Prevotella* spp. in the onset of obesity and obesity-related AN in future studies and clinical practice.

## Data Availability

The raw sequence data reported in this study have been deposited in the Genome Sequence Archive [1] in the National Genomics Data Center [2], China National Center for Bioinformation/Beijing Institute of Genomics, Chinese Academy of Sciences (GSA: CRA011157), that are publicly accessible at https://ngdc.cncb.ac.cn/gsa.

## Introduction

Obesity, one side of the double burden of malnutrition, is one of the most serious global public health challenges. Over half of Chinese adults were found to be overweight or obese in the most recent national survey [3]. Obesity is a chronic, relapsing, progressive medical condition and can cause premature disability and death by increasing the risk for multiple noncommunicable diseases [3,4]. Thus, reducing the burden of obesity and obesity-related disease on health and society and reversing the increase in obesity prevalence are high priorities worldwide.

To date, health recommendations depend on the fact that the fundamental cause of obesity is an energy imbalance between calories consumed and expended. However, systemic forces and environmental determinants are not the only drivers of obesity; genetic susceptibility, psychosocial factors, obesogens and adverse early-life exposures are amongst other concurrent potential risk factors [3]. Recent evidence has indicated that the human microbiota (mainly the gut microbiota) plays a central role in obesity and may serve as a potential innovative biomarker for obesity [5,8]. Our previous work also suggested that various degrees of obesity involve different gut microbial spectra and that inhibiting certain gut microbiota species in different obesity groups may be an important way to improve obesity [9]. The oral cavity, the beginning of the digestive system, physically connects with the intestine. Same as gut microbiota, oral microbiota also plays a vital role in diverse health conditions; it has been shown that oral microbiota connects complexly and importantly with gut microbiota by the oral–gut microbiome axis [10,12]. The oral microbiota has been shown to affect body health via the digestion of specific foods [13,14]. Furthermore, evidence linking the oral microbiota to a myriad of extra-oral diseases including obesity and overall health keeps on accumulating [15,18]. However, the oral microbiota, an important aspect of the human microbiota, has yet to be studied extensively in obesity and obesity-related comorbid disorders.

Acanthosis nigricans (AN) is characterized by a velvety appearance, papillose thickening and brownish-black pigmentation of the epidermis, typically affecting the skin in areas exposed to friction [19]. Obesity is the most common cause of AN serving as a cutaneous marker of insulin resistance [20,21]. It is increasingly observed in children and adolescents with obesity. The prevalence of AN was 5.7%–9.3% at the age of 11 to 16 and commonly occurred in the adult population [22,23]. AN treatments have not been extensively studied, although various treatment options, including oral, topical and chemical peels, have been assessed in smaller powered clinical trials and case reports [24,25]. Till now, rare studies focus on the connection between microbiota and AN. Gao *et al*. reported that patients with obesity and AN had worse gut microbiota [7]. Recently, the vaginal microbiome was shown to associate with specific clinical manifestations of polycystic ovary syndrome including AN [26]. However, the differential distribution of the oral microbiota and its potential association with metabolic markers of obesity-related AN have rarely been investigated.

In this context, we hypothesized that the oral microbiota might be a target for preventing, diagnosing and treating obesity and obesity-related AN. Therefore, we performed a large-scale, 16S rRNA gene high-throughput sequencing-based oral microbiota analysis to evaluate the altered composition and functions of the oral microbiota in obesity with or without AN. Additionally, we evaluated the ability of examining oral microbiota to differentiate obese individuals with or without AN from normal controls. Our results demonstrated the relationship between oral microbiota alterations and obesity-related AN and shed light on potential microbiota-focused strategies for preventing or treating this disease.

## Methods

### Study participants, design and sample collection

We studied the saliva microbiota from 149 participants: 50 healthy individuals (control group), 49 individuals with obesity (obesity group) and 50 individuals with obesity and AN (Ob_AN group). Weight and height were measured for each participant. Body mass index (BMI) was calculated as weight (kg) divided by height (m^2^). All patients with obesity (BMI ≥28 kg m^−2^) were hospitalized before bariatric surgery at the Third People’s Hospital of Chengdu, Chengdu, China. The people with obesity were participants of the Longitudinal Study of Bariatric Surgery in Western China registered at the Chinese Clinical Trial Registry, number ChiCTR2300073353 (https://www.chictr.org.cn/showprojEN.html?proj=175254). The control participants (18.5<BMI<24 kg m^−2^) were healthy volunteers matched for age and sex. Saliva samples were transported to the laboratory immediately after collection, preprocessed and stored at −80 °C. In brief, ~2 ml whole saliva samples were collected from people between meals by sterile vessels (non-stimulated). After centrifugation (16,000 ***g***, 4 °C, 15 min), salivary pellet in one sterile tube and supernatant in the other sterile tube were separated and stored. The pellet was subjected to microbial DNA extraction and sequencing and then compared to obtain a profound understanding of the saliva microbiota of obesity and obesity-related AN patients.

### Microbial DNA extraction and sequencing

Total genomic DNA was extracted from saliva samples using the MagPure Soil DNA LQ kit (Magen Biotech, Guangzhou, China). DNA concentration and purity were monitored using a NanoDrop 2000 spectrophotometer (Thermo Fisher Scientific, Waltham, MA, USA) and agarose gel electrophoresis. DNA samples were diluted to 1 ng µl^−1^ and stored at −20 °C until further use. For sequencing, the 16S rRNA gene V3–V4 region of bacteria was firstly amplified using a specific primer (343F: TACGGRAGGCAGCAG; 798R: AGGGTATCTAATCCT) with unique barcodes (PCR procedure: 94 °C/5 min, 1 cycle; 94 °C/30 s, 56 °C/30 s and 72 °C/20 s, 26 cycles; 72 °C/5 min, 1 cycle; 4 °C, hold). The amplicon quality was visualized using gel electrophoresis. Then, the PCR products were purified with Agencourt AMPure XP beads (Beckman Coulter Co., USA) and amplified for another round of PCR (PCR procedure: 94 °C/5 min, 1 cycle; 94 °C/30 s, 56 °C/30 s and 72 °C/20 s, 7 cycles; 72 °C/5 min, 1 cycle; 4 °C, hold). After being purified with the AMPure XP beads again, the final amplicon was quantified using a Qubit dsDNA assay kit. Equal amounts of purified amplicon were pooled for subsequent sequencing. Finally, the 16S sequencing was performed on an Illumina NovaSeq6000 with two paired-end read cycles of 250 bases each (Illumina Inc., San Diego, CA; OE Biotech Company; Shanghai, China).

### Microbial data processing and analysis

Microbial reads sequenced were processed and analysed using QIIME 2 (version 2020.11) [27]. Raw data were demultiplexed and quality filtered using the q2-demux plugin, followed by denoising with DADA2 (via q2‐dada2) [28]. The generated amplicon sequence variants (ASVs) were aligned with mafft (via q2-alignment) and used to construct a phylogenetic tree with FastTree2 (via q2-phylogeny) [29,30]. The silva database (version 138) was used to align with the sequences of ASVs (via q2-feature-classifier) for the bacterial taxonomic assignment and identification [31,32]. The microbial diversity in saliva samples was estimated using the alpha diversity, including the Chao1 [33] and Shannon [34] indices. The Bray–Curtis, Jaccard and unweighted UniFrac distance matrices performed with QIIME2 were used for principal coordinate analysis (PCoA). The Phylogenetic Investigation of Communities by Reconstruction of Unobserved States (PICRUSt2) (v2.5.0) workflow was applied to predict the metagenome functions of the microbiota with default parameters [35]. A stamp plot was generated using OmicStudio tools (https://www.omicstudio.cn/tool) [36].

### Statistical analysis

The differences in alpha and beta diversity amongst groups were separately compared using ANOVA with Tukey HSD correction and permutational multivariate ANOVA (permutation=999). Linear discriminant analysis (LDA) effect size (LEfSe) was employed for the microbial biomarker analysis. The LDA score cutoff was set to 3.0 in the LEfSe biomarker analysis. The receiver operating characteristic (ROC) curve of microbial biomarkers was used to evaluate the diagnostic value of the oral microbiota. The Kruskal–Wallis test was used to assess the differences in predicted functional outcomes between the groups. The significance cutoff was set as *P*<0.05.

## Results

### Baseline characteristics of participants with obesity

There were no significant differences in sex, age and height between the Ob_AN and obesity groups. However, weight, BMI, percent fat-free mass of the trunk, visceral fat area, arm circumference, arm muscle circumference, fat-free mass index, fat mass index and the measured circumference of the neck, chest, abdomen, hip, right arm, left arm, right thigh and left thigh were significantly higher in the Ob_AN group than that in the obesity group (Table 1).

**Table 1.** Baseline characteristics of the people with obesity or obesity with AN

	Ob_AN	Obesity	*P* value
Male/female	17/33	17/32	0.48
Age (year)	29.85±6.4336	32.87±8.2995	0.056
Height (cm)	163.57±6.7656	164.24±9.081	0.631
Weight (kg)	104.86±20.1998	96.14±20.6592	0.022
BMI (kg m^−2^)	38.96±5.6818	35.50±6.0749	< 0.001
PBF (percent body fat)	46.17±5.6443	44.10±6.2767	0.11
FFM (fat-free mass) of trunk (kg)	26.2±4.8583	24.67±4.526	0.089
FFM% of trunk	107.09±7.13	103.11±4.7525	0.002
WHR (waist-hip ratio)	1.02±0.0563	0.99±0.0606	0.022
VFL (visceral fat level)	18.94±1.9193	18.15±2.4925	0.052
VFA (visceral fat area, cm^2^)	219.53±40.7872	195.33±38.3975	0.003
AC (arm circumference) (cm)	42.03±5.8394	39.33±6.2243	0.003
AMC (arm muscle circumference) (cm)	33.29±4.3129	31.57±4.6528	0.007
FFMI (fat-free mass index)	20.82±2.6694	19.63±2.4546	0.022
FMI (fat mass index)	18.15±4.2618	15.88±4.5566	0.006
Measured circumference of neck (cm)	43.59±3.749	42.13±5.6411	0.01
Measured circumference of chest (cm)	114.4±8.5951	109.71±9.6321	0.005
Measured circumference of abdomen (cm)	119.73±13.5853	111.62±11.7779	0.004
Measured circumference of hip (cm)	117.2±8.5025	112.66±9.4975	0.003
Measured circumference of right arm (cm)	42.37±5.9141	39.50±6.3456	0.002
Measured circumference of left arm (cm)	42.03±5.8394	39.33±6.2243	0.003
Measured circumference of right thigh (cm)	64.13±4.9844	62.08±6.5007	0.015
Measured circumference of left thigh (cm)	62.95±4.6106	60.92±5.4932	0.015
SMI (skeletal muscle index)	8.65±1.1669	8.26±1.2443	0.099

Ob_AN: people with obesity and AN. An independent samples t-test (Mann–Whitney U) was used. Value was presented by mean±sd.

### Richness and diversity of oral microbiota in obesity with/without AN

**Fig. 1.**
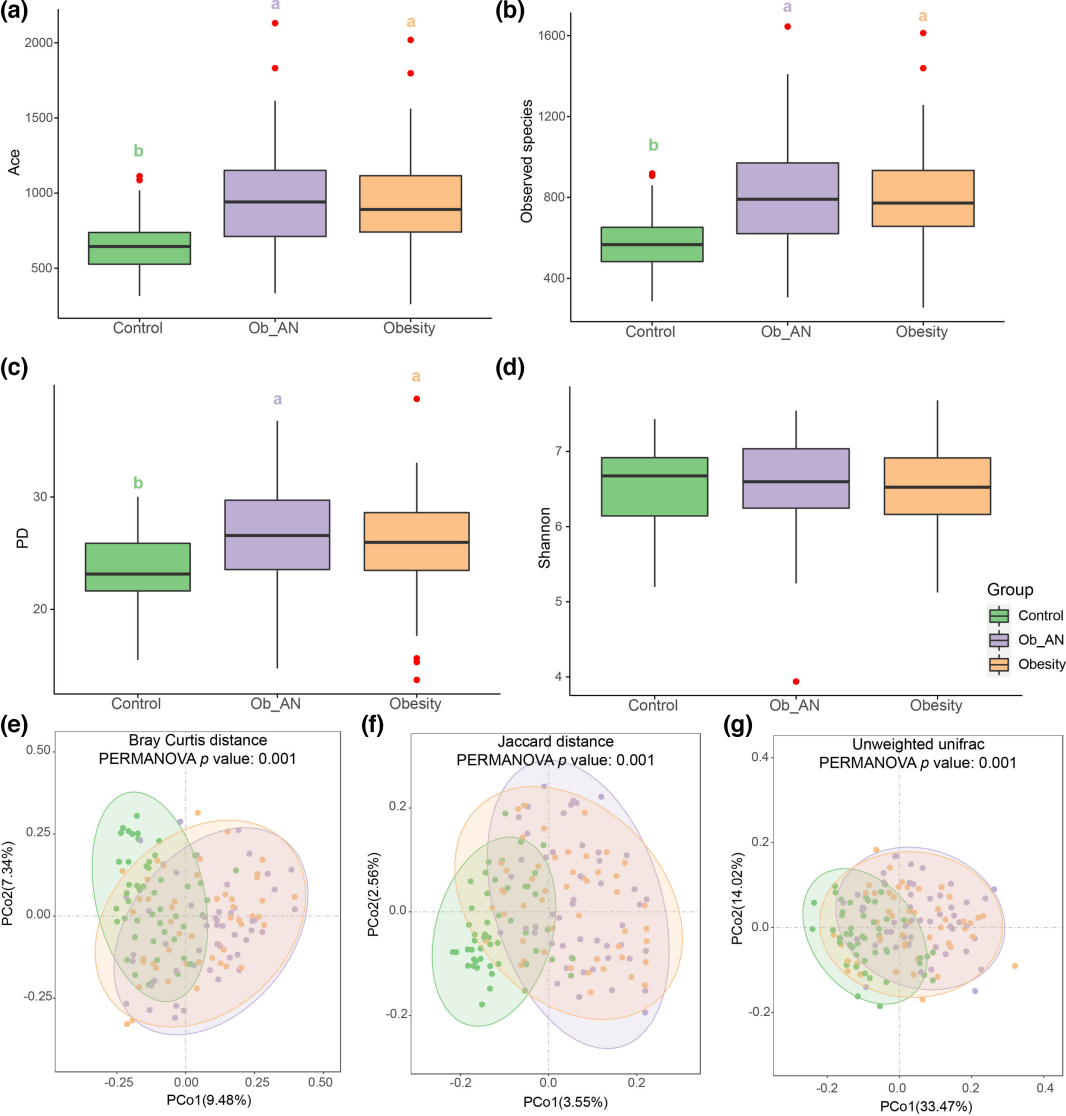
Oral microbial diversity in patients with obesity or obesity with AN. (a–d) Microbial alpha diversity was estimated based on ACE (**a**), observed species (**b**), Faith’s PD (**c**) and Shannon (**d**) indices in the healthy control (*n*=50), obesity (*n*=49) and obesity with AN (Ob_AN, *n*=50) subgroups. Different lowercase letters indicate significant differences (*P*<0.05). (e–g) Beta diversity of Bray–Curtis (**e**), Jaccard (**f**) and unweighted UniFrac (**g**) distances in the three groups based on PCoA. Different lowercase letters in a–d indicated significant differences (*P*<0.05).

We first assessed the microbial richness [abundance-based coverage estimators (ACEs) and observed species indices] and diversity [Faith’s phylogenetic diversity (PD) and Shannon indices] in saliva samples from this study population (Fig. 1). No differences in the richness and diversity were found between the Ob_AN and obesity group samples. However, the ACE (Fig. 1a), observed species (Fig. 1b) and PD diversity (Fig. 1c) indices were significantly higher in the Ob_AN and obesity groups than in the control group. In contrast, no differences in the Shannon index were observed between the two obesity groups and the control (Fig. 1d). Similarly, the microbial beta diversity demonstrated distinct separation in the microbial community amongst the three groups as shown using PCoA of Bray–Curtis (Fig. 1e), Jaccard (Fig. 1f) and unweighted UniFrac distances (Fig. 1g). In addition, the oral microbiota of the Ob_AN group was similar to that of the obesity group. These results imply that significant dysbiosis arises in the oral microbiota in obesity with or without AN; differences in weight and BMI of obesity did not affect the oral microbial diversity in the current study.

### Variations in the composition of oral microbiota in different groups

**Fig. 2.**
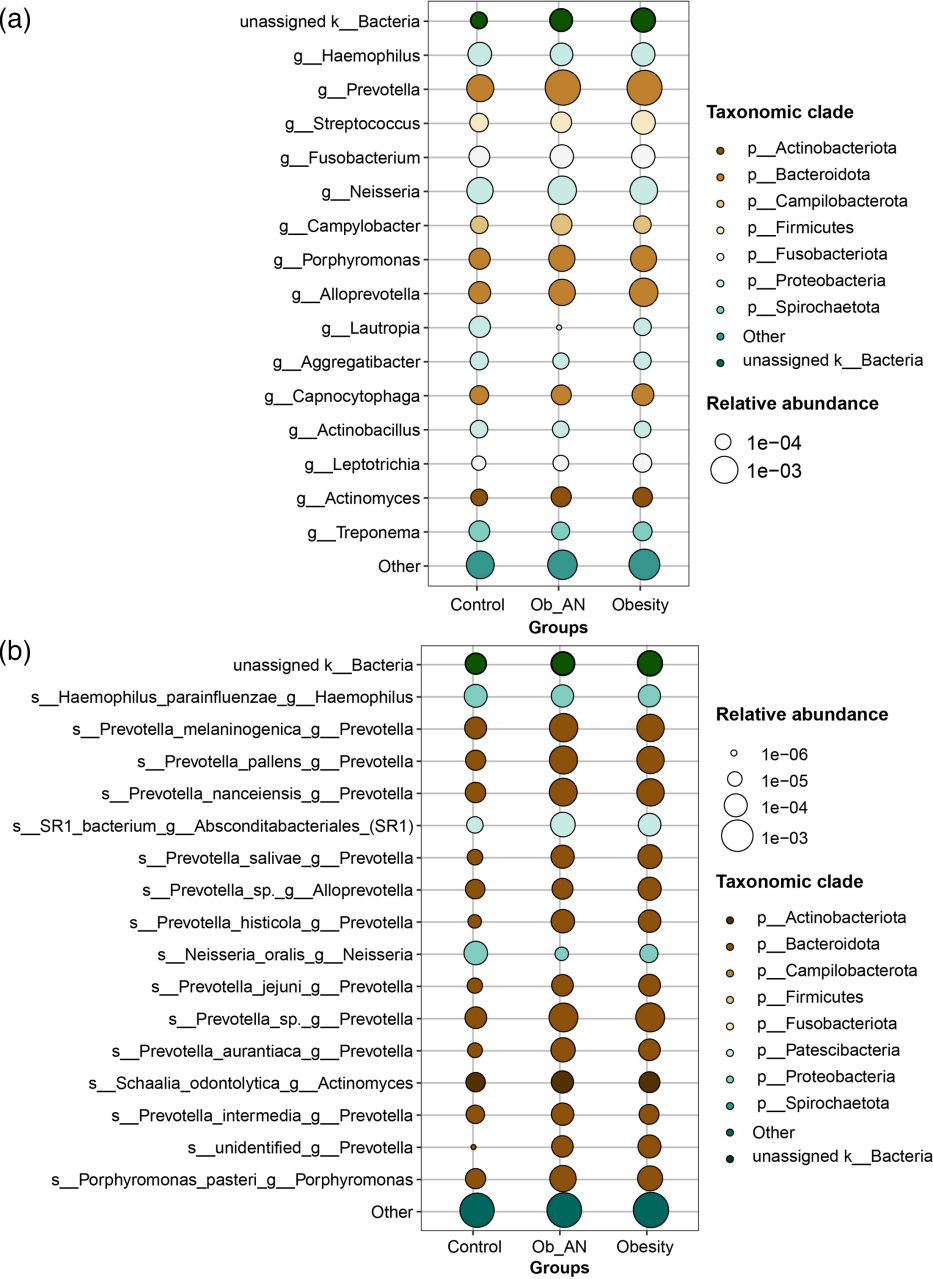
Oral microbial composition in patients with obesity or obesity with AN. (**a**) Top 15 genera in the healthy control, obesity and obesity with AN (Ob_AN) subgroups at the ASV level. (**b**) The top 16 taxonomic species are shown for the three groups at the ASV level.

In total, 17 phyla, 31 classes, 76 orders, 136 families, 283 genera and 686 species were assigned. At the phylum level, Bacteroidota (control: 28.28%; Ob_AN: 44.31%; obesity: 41.22%), Pseudomonadota (Proteobacteria, 40.93%, 28.87% and 28.61%), Bacillota (Firmicutes, 11.86%, 11.06% and 13.23%), Fusobacteriota (9.87%, 7.08% and 8.35%), Actinomycetota (Actinobacteriota, 2.91%, 3.15% and 3.70%) and Campilobacterota (2.52%, 2.25% and 2.10%) were predominant in all three groups (Fig. S1A, available in the online Supplementary Material). At the genus level, *Prevotella* (control: 12.21%; Ob_AN: 28.97%; obesity: 24.64%) genus was most abundant in all three groups, followed by *Neisseria* (17.70%, 14.83% and 12.80%), *Haemophilus* (14.16%, 9.69% and 11.30%), *Fusobacterium* (8.05%, 6.00% and 6.56%), *Alloprevotella* (5.32%, 6.33% and 8.32%), *Streptococcus* (5.52%, 5.27% and 7.60%), *Porphyromonas* (5.89%, 6.35% and 5.01%), *Campylobacter* (2.50%, 2.24% and 2.09%), *Capnocytophaga* (2.57%, 1.60% and 2.15%) and *Aggregatibacter* (2.65%, 1.11% and 1.34%) (Figs 2a and S1B). At the species level, the top 10 species were *Haemophilus parainfluenzae* (control: 8.84%; Ob_AN: 6.31%; obesity: 7.03%), *Prevotella melaninogenica* (3.16%, 10.50% and 8.04%), *Fusobacterium periodonticum* (3.45%, 3.67% and 4.19%), *Prevotella nanceiensis* (1.08%, 3.54% and 2.87%), *Prevotella pallens* (0.83%, 3.49% and 3.04%), *Fusobacterium nucleatum* (3.66%, 1.69% and 1.62%), *SR1 bacterium* (0.99%, 2.00% and 1.53%), *Prevotella* sp. (1.24%, 1.49% and 1.52%), *Campylobacter concisus* (1.16%, 1.57% and 1.51%) and *Porphyromonas gingivalis* (2.30%, 0.40% and 0.73%) (Fig. S2). Moreover, at the ASV level, many *Prevotella* spp., including *P. melaninogenica*, *P. pallens* and *P. nanceiensis*, were identified and enriched in the Ob_AN and obesity groups (Fig. 2b).

**Fig. 3.**
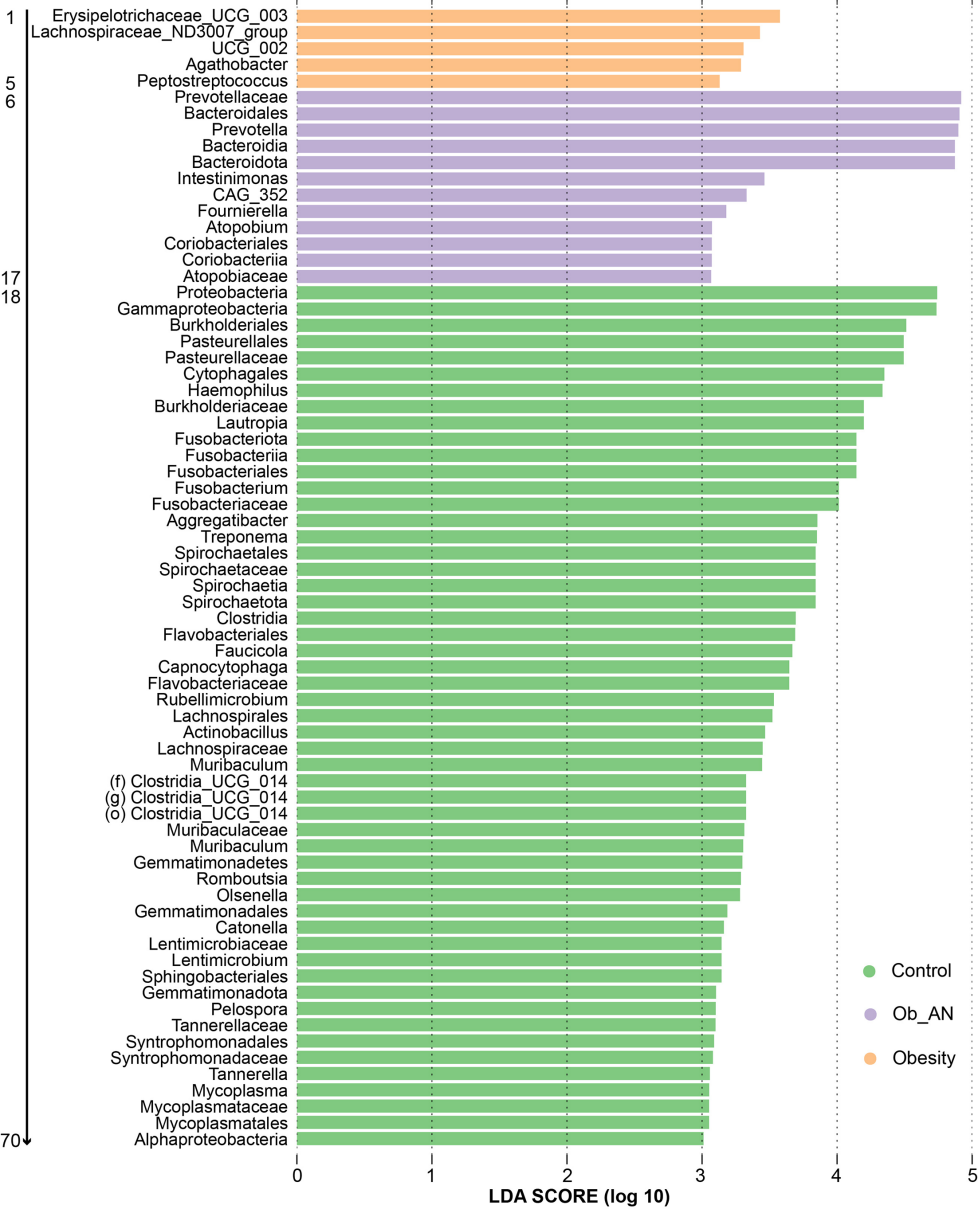
Microbial biomarker analysis between groups using LEfSe. The numbers correlate with Table S1 and indicate the taxonomic name of the related taxa. A total of 5, 12 and 53 taxa were biomarkers in the obesity, obesity with AN (Ob_AN) and control groups, respectively. LDA score (log 10) cutoff>3.0.

Furthermore, LEfSe was employed to identify the key microbiota associated with disease and health to determine the essential microbiota constituting the oral microbial community. *Erysipelotrichaceae UCG 003*, *Lachnospiraceae ND3007 group*, *Oscillospiraceae UCG 002*, *Agathobacter*, *Peptostreptococcus*, Prevotellaceae, *Prevotella*, Bacteroidota, Bacteroidia, Bacteroidales, *Intestinimonas*, *CAG 352*, *Fournierella*, *Atopobium*, Coriobacteriia, Coriobacteriales and Atopobiaceae were the key microbes associated with microbial dysbiosis in the obesity and Ob_AN groups. Fifty-three taxa, including Proteobacteria, *Haemophilus*, *Lautropia*, *Fusobacterium*, *Aggregatibacter*, *Muribaculum*, Lachnospiraceae and *Muribaculaceae* at the genus level, were the key microbiota associated with a healthy/non-obese state (Fig. 3 and Table S1).

**Fig. 4.**
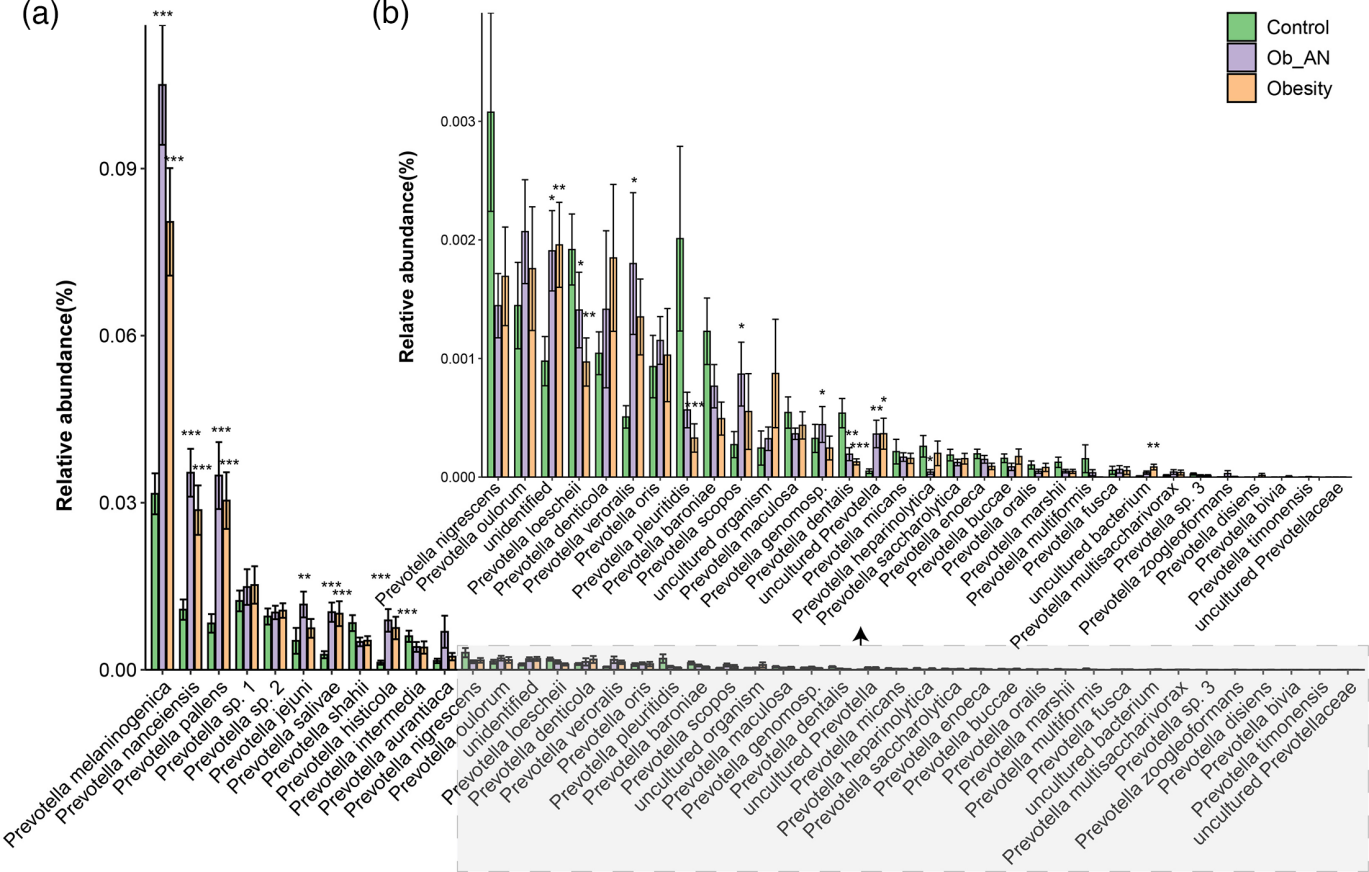
Varieties of *Prevotella* spp. in the oral cavity of patients with obesity or obesity with AN. (**a**) A total of 43 species were identified. (**b**) Replot of species *P. nigrescens* to *uncultured Prevotellaceae* in panel (**a).** A Kruskal–Wallis test was used. *P<0.05, **P<0.01 and ***P<0.001 when compared to the control group.

Subsequently, we focused on *Prevotella* spp., which were significantly enriched in the obesity and Ob_AN groups. A total of 43 species were identified, and most were abundant in the obesity and Ob_AN groups, especially in the Ob_AN group, such as high-abundance taxa *P. melaninogenica*, *P. nanceiensis* and *P. pallens* (Fig. 4). Low-abundance *Prevotella* spp., including *Prevotella shahii*, *Prevotella intermedia*, *Prevotella nigrescens*, *Prevotella loescheii*, *Prevotella pleuritidis*, *Prevotella baroniae* and *Prevotella dentalis*, were abundant in the control group (Fig. 4). Altogether, the results indicate that a notable reduction of many oral microbiota components (very low abundance) and a high level of *Prevotella* spp. may be associated with AN in obesity.

### Functional prediction in obesity with/without AN using PICRUSt2

**Fig. 5.**
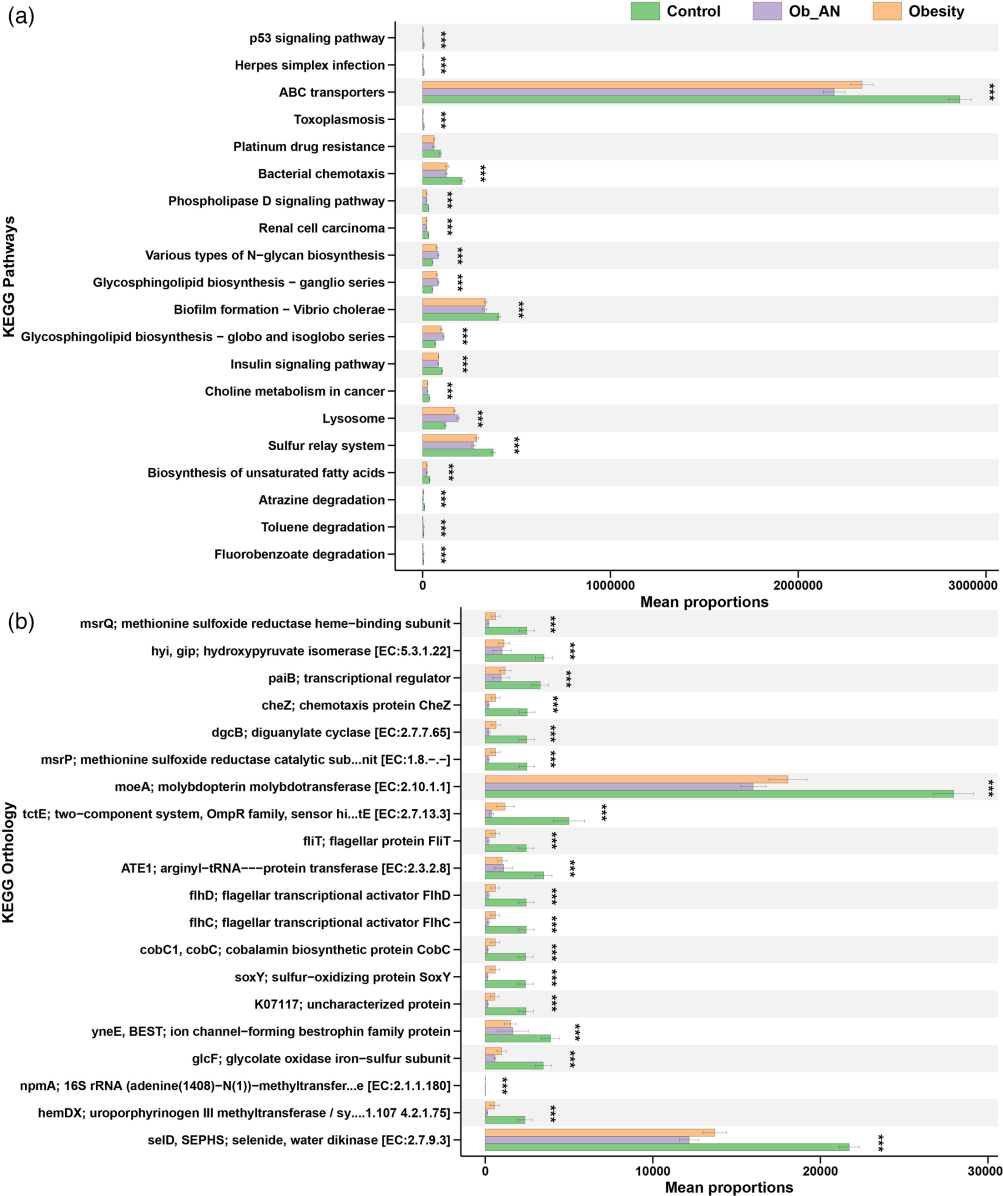
Alterations in the predicted metagenome functions in different groups. The top 20 significant KEGG pathways (**a**) and orthologues (**b**) are shown. A Kruskal–Wallis test was used. ***P<0.001 amongst three groups.

We conducted a functional prediction using the PICRUSt2 pipeline based on the Kyoto Encyclopedia of Genes and Genomes (KEGG) database to evaluate functional alterations in the oral microbiota in obesity with or without AN. The most abundant gene functions in the oral microbiota community were related to metabolism (especially amino acid and lipid metabolism) and membrane transport (Figs S3 and S4). On further analysis, we identified significantly changed microbial functions at category level 3 in the three groups (Fig. 5). In the disease groups (Ob_AN and obesity), the dominant altered function of the microbiome was the downregulation of ATP-binding cassette (ABC) transporters (Fig. 5a). However, genes related to lysosome and glycosphingolipid biosynthesis – globo and isoglobo series – showed a significant increase (Fig. 5a). In the analysis of KEGG Orthology, abundance levels of genes linked to molybdopterin molybdotransferase [moeA; EC:2.10.1.1] and selenide, water dikinase [selD, SEPHS; EC:2.7.9.3] decreased significantly (Fig. 5b).

**Fig. 6.**
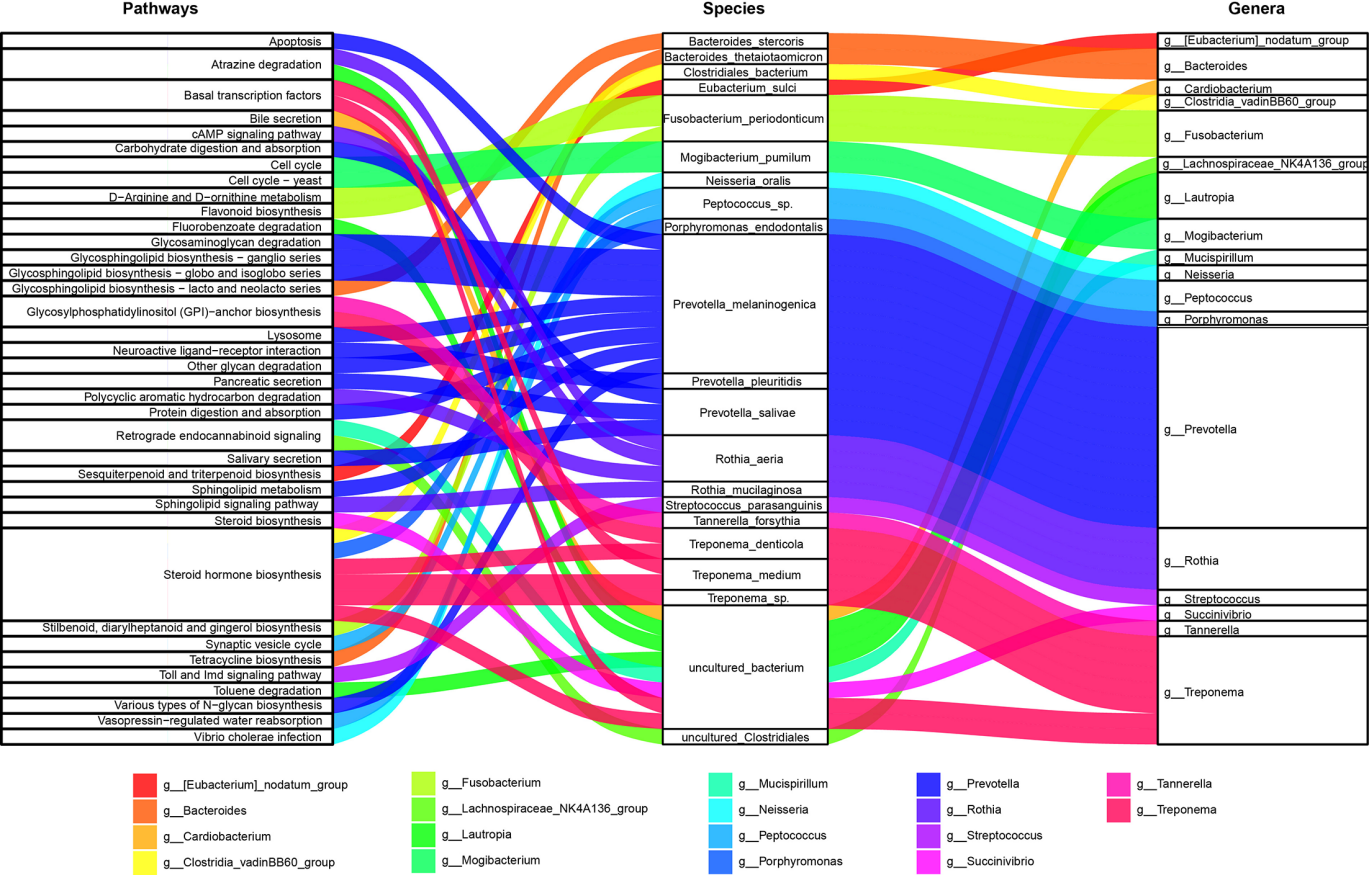
Sankey diagram of predicted pathways and microbial species. A Spearman correlation analysis was performed; *rho* cutoff >0.7. Left nodes: predicted pathways; middle nodes: microbial species; right nodes: species-related microbial genera. The flows indicated that the pathways were positively linked to microbiota.

Furthermore, we assessed which microbiota species were associated with these functions using Spearman’s correlation and network analyses. Genus *Prevotella* was found to contribute substantially (Figs 6 and S5 and Table S2). *P. melaninogenica* was significantly correlated with apoptosis, glycosaminoglycan degradation, glycosphingolipid biosynthesis, lysosome, sphingolipid metabolism and protein digestion and absorption. *P. pleuritidis* was correlated with neuroactive ligand−receptor interaction. *Prevotella salivae* was linked with carbohydrate digestion and absorption and pancreatic and salivary secretion. Together, these results demonstrate that significant dysbiosis in oral microbiota functions may be associated with obesity with or without AN, and *Prevotella* spp. may play an important role in obesity and AN.

### Distinguishing amongst the three groups based on oral microbiota

**Fig. 7.**
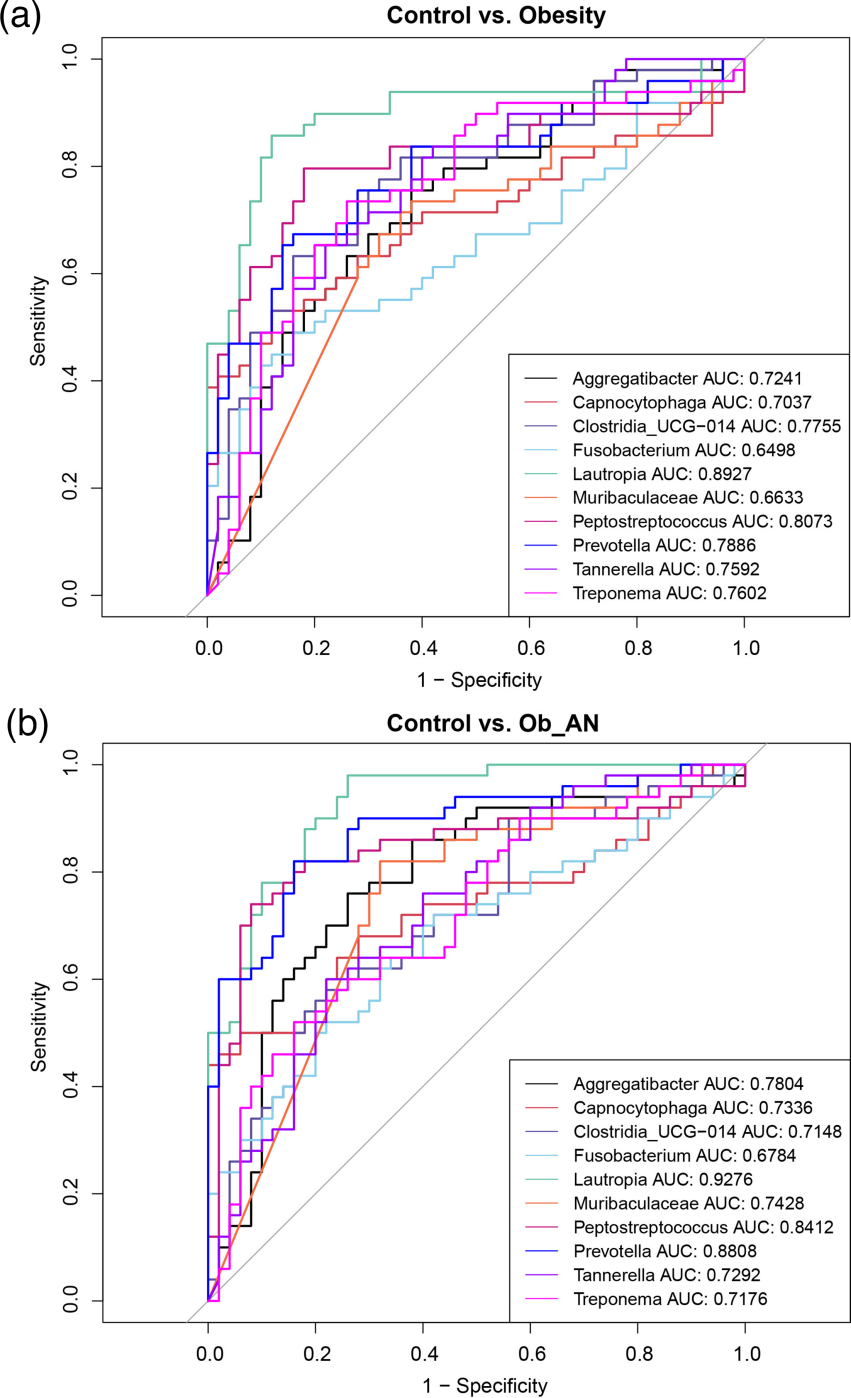
Evaluation of the distinguishable value of oral microbiota through ROC curve analysis. The top 10 genera that can effectively distinguish obesity (**a**) or obesity with AN (Ob_AN) samples (**b**) from healthy control samples are shown.

Finally, we chose microbiota components differentially altered in the three groups at the genus level (determined by LEfSe) to distinguish the Ob_AN or obesity groups from the control group through ROC curve analysis. Ten taxa, *Aggregatibacter*, *Capnocytophaga*, *Clostridia*, *Fusobacterium*, *Lautropia*, *Muribaculaceae*, *Peptostreptococcus*, *Prevotella*, *Tannerella* and *Treponema*, were found to serve as candidates; however, these taxa could not effectively distinguish obesity from Ob_AN (Figs 7 and S6). *Lautropia* (AUC=0.8927) and *Peptostreptococcus* (AUC=0.8073) were the top two taxa in the obesity-alone group, followed by *Prevotella* (AUC=0.7886) (Fig. 7a). *Lautropia* (AUC=0.9276) and *Prevotella* (AUC=0.8808) were the dominant genera in the Ob_AN group (Fig. 7b). The results indicate that oral microbiota components could be biomarkers for diagnosing obesity with or without AN.

## Discussion

Given the worldwide prevalence of obesity, this disease demonstrates pandemic proportions [4,37,39]. The gut microbiota has been demonstrated to directly regulate host energy metabolism and obesity using germ-free animal models [40,41]. However, it remains unknown which specific bacteria or group of bacteria can predict the onset of obesity or body weight loss in humans. To date, numerous studies have been conducted to explore oral microbiota alterations in obesity, most of which have focused on children and adolescents [42,45]. In the current study, we sequenced 16S rRNA amplicons of oral bacteria from participants with obesity with or without AN and healthy controls. Marked dysbiosis with altered bacterial diversity, abundance and potential functions was seen in patients with obesity and obesity-related AN. To the best of our knowledge, for the first time, we provide important evidence that oral microbiota dysbiosis might be associated with obesity with or without AN.

Consistent with the findings of a previous study, the microbial richness (ACE index) in saliva samples in the disease (Ob_AN and obesity) groups was significantly greater than that in the control group [42,44]. Although higher microbial richness (such as numbers of ASVs) was observed in samples from the disease groups versus the control group, the three groups showed similar Shannon diversity, likely due to the high proportion of *Prevotella* in the disease group samples (Figs2b  4). Notably, Bacteroidota, Pseudomonadota, Bacillota, Fusobacteriota, Actinomycetota and Campilobacterota were the dominant phyla regardless of condition [44]. Interestingly, *P. melaninogenica*, *P. pallens* and *P. nanceiensis* were more abundant in the disease groups than in the healthy control group. *P. pallens* is a typical species of the *Prevotella* organism [46]. In addition, *P. nanceiensis* could have Toll-like receptor 2-dependent inflammatory effects [47]. Furthermore, Pedersen *et al*. have suggested that *Prevotella* spp. could induce obesity-related metabolic disorders including insulin resistance and glucose intolerance [48]. Together, oral *Prevotella* spp. are linked to inflammation and insulin resistance – well-known contributors to metabolic disorders, including obesity and obesity-related AN.

Importantly, numerous taxa, including *Haemophilus*, *Lautropia*, *Fusobacterium*, *Aggregatibacter*, *Muribaculum* and Lachnospiraceae, were absent in the disease groups versus the healthy control group. *Lautropia* spp. are primarily involved in energy metabolism, such as the TCA cycle, pyruvate metabolism, glycolysis and fatty acid metabolism [49]. The clinical significance of *Haemophilus* and *Aggregatibacter* has been well reviewed by Nørskov-Lauritsen, who associated specific species with various clinical syndromes [50]. *Muribaculum* and Lachnospiraceae, frequently reported in the gut, are considered potentially beneficial. Indeed, accumulating evidence has implied that diseases such as obesity are associated with microbial changes, especially the reduction of some key taxa (referred to as microbiota dysbiosis). Additionally, the substantial increase in *Prevotella* spp. levels could inhibit the growth of the other microbiota in the limited oral environment. Thus, significant oral bacterial dysbiosis is closely associated with obesity and obesity-related AN.

In humans, ABC transporters, one of the largest known protein families, have been confirmed to be closely related to the pathogenesis of diseases such as obesity due to their ability to regulate lipid metabolism [51]. Specifically, ABC transporters are involved in cholesterol homeostasis [52]. The ABC transporters downregulated in obesity and obesity-related AN patients in the current study are also widespread in bacteria. These transporters are vital virulence factors in bacteria due to their role in nutrient uptake and toxin and antimicrobial agent secretion [53]. Given the decrease in ATP-driven ABC transporters, we also found a marked decrease in molybdopterin molybdotransferase [moeA; EC:2.10.1.1], which can produce adenosine monophosphate. One reason for this phenomenon may be that 53 taxa were found to be significantly abundant in the control group, whilst only a small number of taxa [19] were significantly abundant in cases of obesity and obesity-related AN (Fig. 3 and Table S1). Although further analyses revealed that members of the *Prevotella* genus were closely associated with changes in predicted microbial functions, the ABC transporters were not linked to Prevotella spp. (Figs 6 and S5 and Table S2). Notably, *Prevotella* spp. in the present study were found to be dominant in the oral microbiota. *P. melaninogenica*, the most abundant *Prevotella* in this study, was correlated with glycosphingolipid biosynthesis, which is increased in obesity and obesity-related AN patients. *P. melaninogenica* may function in glycosphingolipid biosynthesis by associating with glycan moieties, consequently increasing ceramide molecules [54]. Ceramide levels were elevated in the skeletal muscle, liver and hypothalamus of obese rodents and humans [55]. Furthermore, a study demonstrated that low levels of glycosphingolipids enhance adipocyte function and reduce inflammation in the adipose tissue of obese animals [56]. Therefore, *Prevotella* spp. and increased glycosphingolipid biosynthesis may act as significant periopathogens contributing to obesity and obesity-related AN.

Furthermore, we explored the ability of microbial biomarkers to distinguish obesity and obesity-related AN samples from control samples and predict obesity and obesity-related AN risk. Numerous studies have indicated that microbiota components are powerful biomarkers for predicting health state changes [57]. Several genera, including *Lautropia* and *Prevotella*, were identified and demonstrated substantial potential in identifying obesity and obesity-related AN risk. However, these genera were insufficient to distinguish obesity from obesity with AN. Therefore, more data are required to provide solid evidence and validate these results in the future.

Notably, this study has several limitations. The current study used marker (16S rRNA) gene sequencing for the microbiota analysis to obtain bacterial abundance levels. A metagenome (shotgun sequencing)-combined method or other technologies will improve our results about the minimal differences between obesity with or without AN. Although PICRUSt2 provides powerful marker gene metagenome inference, such amplicon-based microbial inferences require further functional verification [35]. Additionally, combining other omics approaches, including proteomics and metabolomics, will enhance our understanding of the functional association between microbiota and diseases. Diet pattern is a fundamental factor that affects oral microbiota [14]. Thus, detailed dietary information should be considered to uncover any dietary effects. Importantly, oral health, an important factor in shaping oral microbiota, is not considered in this study. Longitudinal investigations and larger cohorts with well-documented disease characteristics and oral health information are also required. Anyway, our results shed light on the importance of oral microbiota in obesity with or without AN. Moreover, substantial evidence exists regarding interactions between the oral and gut microbiota [57,59]. Given the importance of the gut microbiota in obesity and related diseases, integrated analyses of the gut and oral microbiomes are warranted to explore the oral and gut microbiota interactions in obesity-related AN.

## Conclusion

In summary, our findings demonstrate that marked oral microbiota dysbiosis signatures are associated with obesity with or without AN and that *Prevotella* (*P. melaninogenica*) may play a vital role in obesity and obesity-related AN. In addition, oral microbiota analyses may be beneficial in identifying obesity and obesity-related AN risk.

## Supplementary material

10.1099/jmm.0.002020 Uncited Supplementary Material 1.

## Abbreviations

ABC: ATP-binding cassette
ACEs: abundance-based coverage estimators
AN: acanthosis nigricans
ASVs: amplicon sequence variants
BMI: body mass index
KEGG: Kyoto Encyclopedia of Genes and Genomes
LDA: linear discriminant analysis
LEfSe: LDA effect size
PCoA: principal coordinate analysis
PD: phylogenetic diversity
PICRUSt2: Phylogenetic Investigation of Communities by Reconstruction of Unobserved States
