# Supplementary material for: Oral microbiota signatures in obesity with or without acanthosis nigricans in a Chinese cohort
Source: J Med Microbiol. 2025 Jun 4;74(6):002020. doi: 10.1099/jmm.0.002020 (PMC12137914; doi:10.1099/jmm.0.002020)
Supplement: Uncited Supplementary Material 1. [file jmm-74-02020-s001.pdf]

Supporting Information

Oral microbiota signatures in obesity with or without acanthosis nigricans in a Chinese cohort

Yujing Tang[1,2,3#], Qin Li[2,3#], Zhengyun Ren[1,2,3], Nianwei Wu[2,3,4], Hongmei Zhu[2,3,4], Tongtong Zhang[1,2,3,4], Wei Yi[1,2,3], Wantao Ju[1,2,3,5], Yanjun Liu[2,3], Junqing Hu[1,2,3,4]

[1]Obesity and Metabolism Medicine-Engineering Integration Laboratory, Department of General Surgery, The Third People's Hospital of Chengdu, Chengdu, 610031, China.

[2]Center of Gastrointestinal and Minimally Invasive Surgery, Department of General Surgery, The Third People's Hospital of Chengdu, Chengdu, 610031, China.

[3]The Center for Obesity and Metabolic health, Department of General Surgery, The Third People's Hospital of Chengdu, Chengdu, 610031, China.

[4]Medical Research Center, The Third People's Hospital of Chengdu, Chengdu, 610031, China.

[5]School of Life Science and Engineering, Southwest Jiaotong University, Chengdu, 610031, China.

* These authors contributed equally to this work and share first authorship.

Corresponding Authors: Yanjun Liu and Junqing Hu

Email: liuyanjun_001@163.com; hujunqing@swjtu.edu.cn.

Address: No. 82, Qinglong Street, Qingyang District, Chengdu, 610031, China.

Supplemental Figures: Figure S1-S6

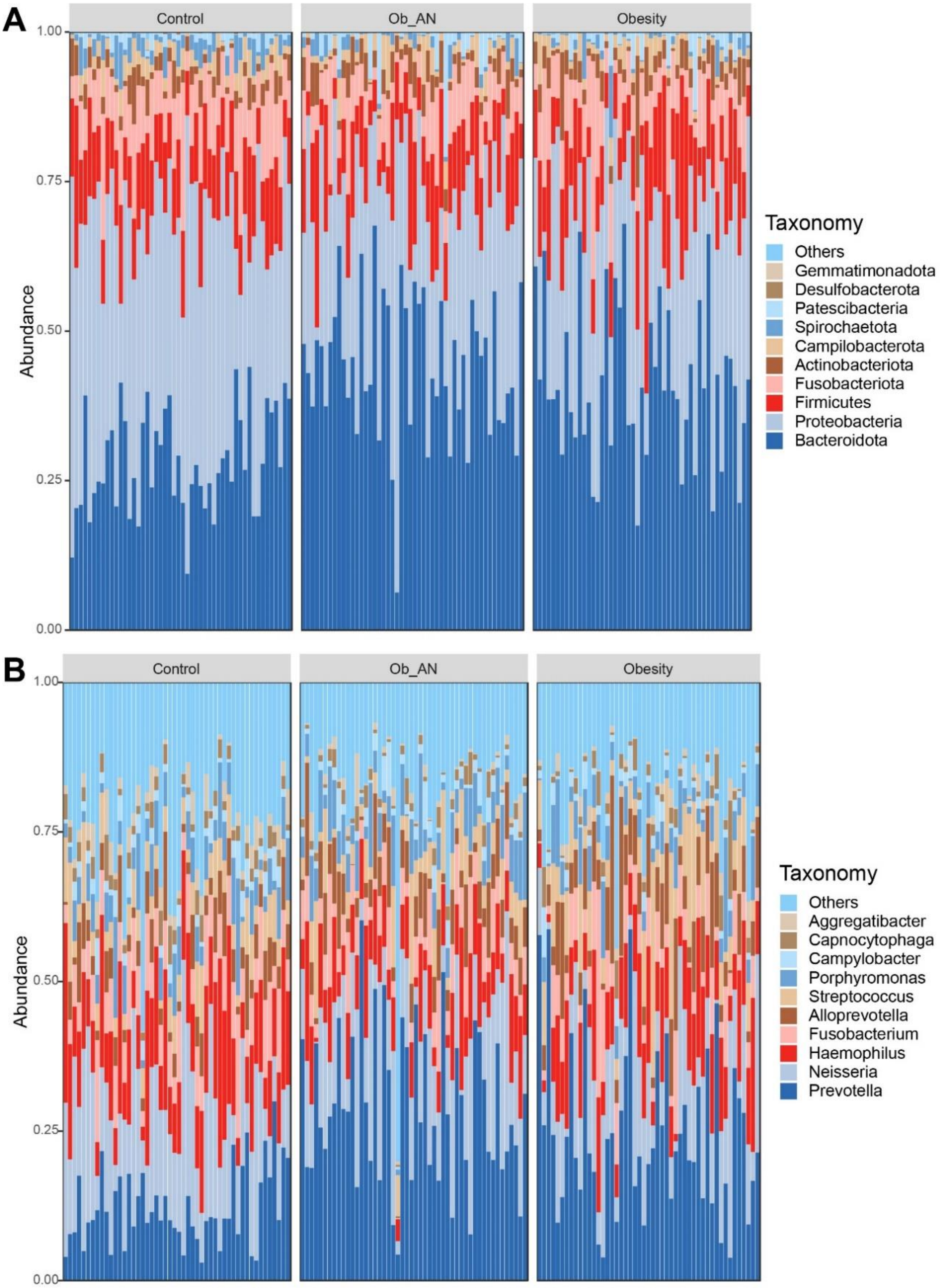

Figure S1 Barplot of oral microbial composition in obesity or obesity with acanthosis nigricans (AN). **A** Top 10 phyla (rank 2) between different groups. **B** Top 10 genera (rank 6) between different groups. Control (n = 50), obesity (n = 49), and obesity with AN (Ob_AN, n = 50).

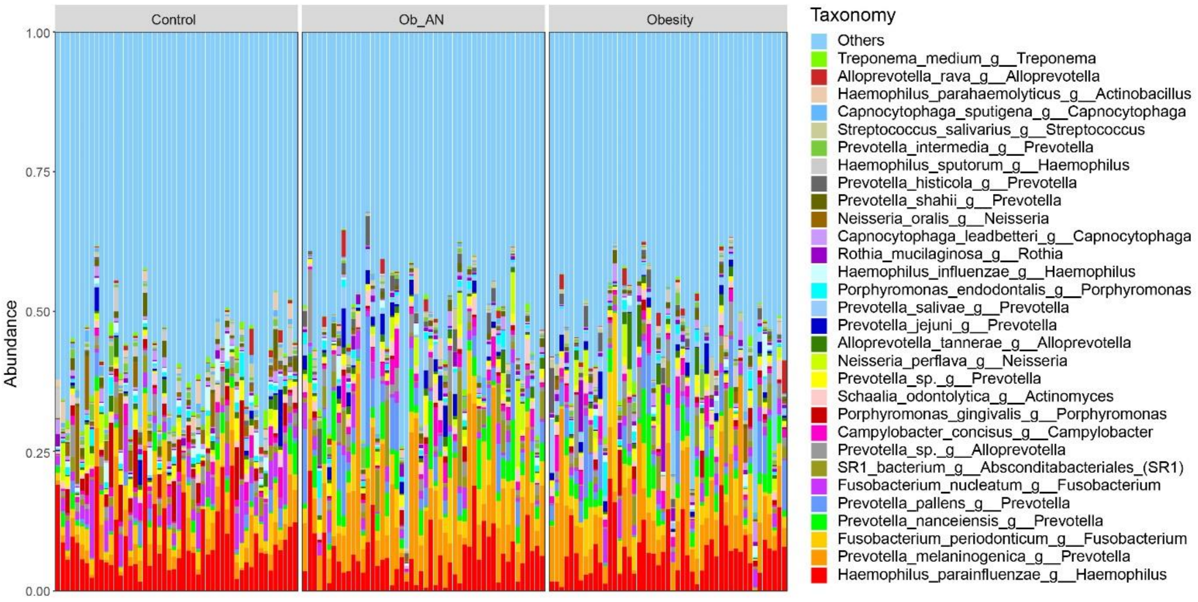

Figure S2 Barplot of oral microbial species (rank 7, species level) in obesity or obesity with acanthosis nigricans (AN). Top 30 bacterial species were shown. Control (n = 50), obesity (n = 49), and obesity with AN (Ob_AN, n = 50).

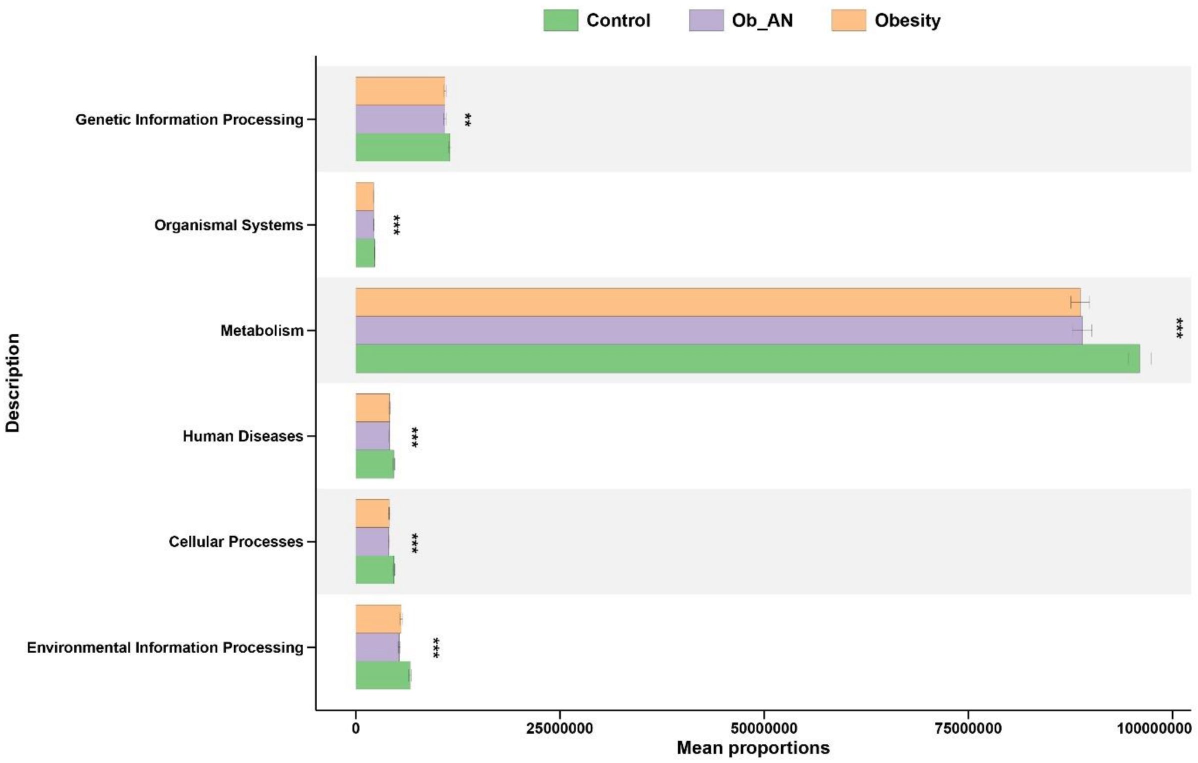

Figure S3 Alterations in the predicted metagenome functions (Level 1) in different groups. A Kruskal-Wallis test was used. ** $p < 0.01$, *** $p < 0.001$ among three groups.

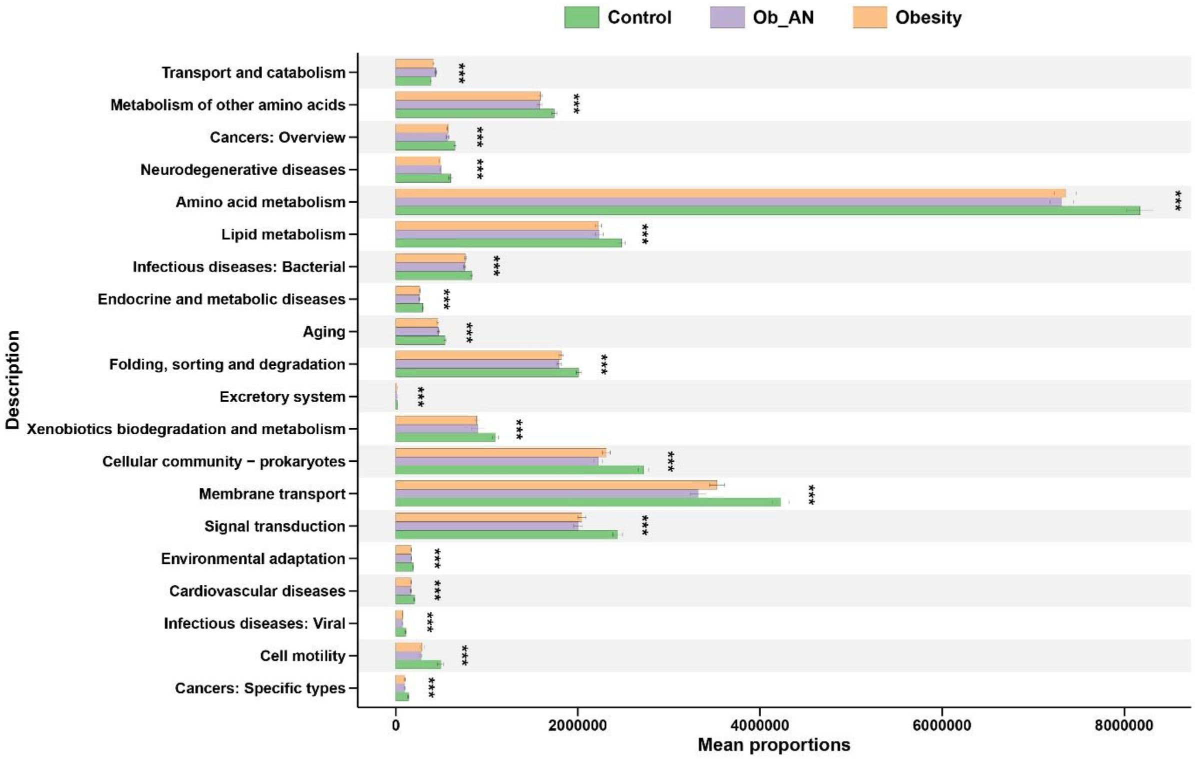

Figure S4 Alterations in the predicted metagenome functions (Level 2) in different groups. Top 20 significant KEGG pathways were shown. A Kruskal-Wallis test was used. *** $p < 0.001$ among three groups.

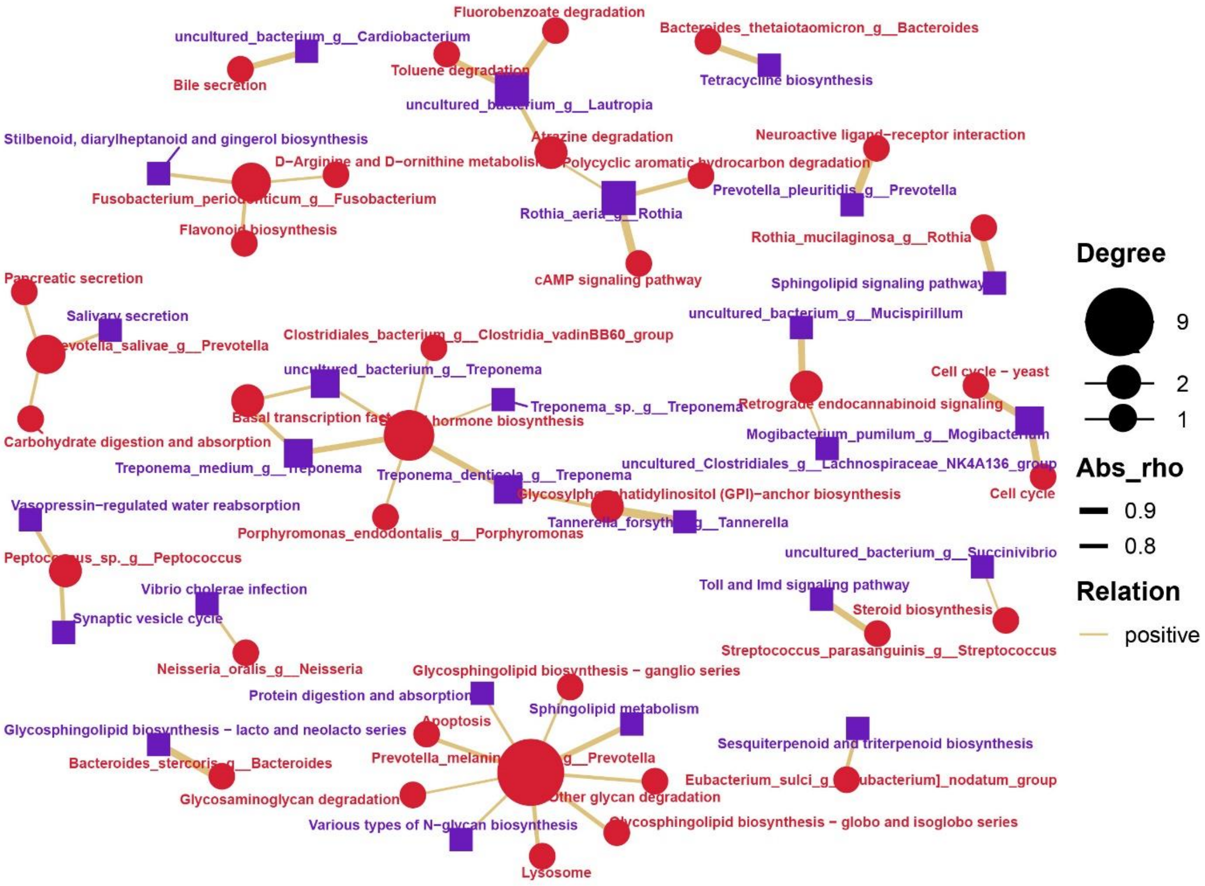

Figure S5 Network of correlation between predicted pathways and microbial species. Correlation was performed by Spearman correlation. *rho* cutoff > 0.7. Node and edge sizes indicate the degree and absolute values of the correlation, respectively. Edges represent the interaction between nodes, and positive correlations are denoted by solid yellow lines.

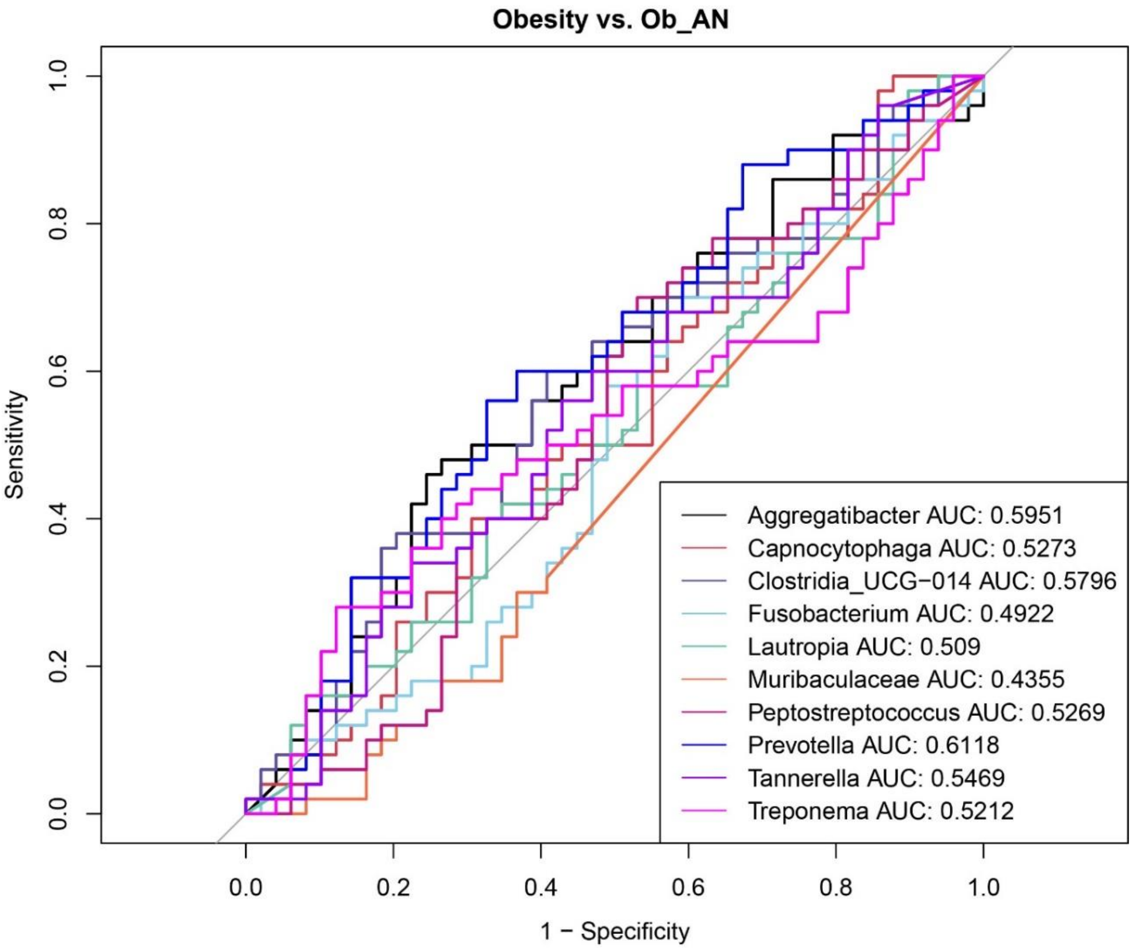

Figure S6 Evaluation of the distinguishable value of oral microbiota between obesity and obesity with AN (Ob_AN) based on the receiver operating characteristic (ROC) curve analysis. Top 10 genera were shown.

Supplemental Tables: Table S1-S2

Table S1 Results of microbial biomarkers analysis across groups by LEfSe method related to Figure 3.

S/N	Biomarker	Logarithm value	Groups	LDA (Log10)	*p*-value
1	g__Erysipelotrichaceae_UCG_003	1.119081029	Obesity	3.576697538	0.038476
2	g__Lachnospiraceae_ND3007_group	1.370465797	Obesity	3.428807695	6.15E-05
3	g__UCG_002	1.912436092	Obesity	3.306994689	0.031363
4	g__Agathobacter	1.57926679	Obesity	3.288526047	0.001777
5	g__Peptostreptococcus	3.557941773	Obesity	3.130438044	7.82E-10
6	f__Prevotellaceae	5.548096293	Ob_AN	4.918921685	4.77E-11
7	o__Bacteroidales	5.624579447	Ob_AN	4.906647847	9.28E-11
8	g__Prevotella	5.461897823	Ob_AN	4.898066498	4.28E-11
9	c__Bacteroidia	5.646594621	Ob_AN	4.872115967	1.64E-10
10	p__Bacteroidota	5.646594621	Ob_AN	4.872110217	1.64E-10
11	g__Intestinimonas	1.266930577	Ob_AN	3.461968141	0.003408
12	g__CAG_352	1.977625323	Ob_AN	3.330014119	2.04E-09
13	g__Fournierella	1.577892265	Ob_AN	3.179621111	6.95E-05
14	g__Atopobium	3.478049646	Ob_AN	3.073815446	5.14E-07
15	o__Coriobacteriales	3.48307545	Ob_AN	3.072469712	1.66E-06
16	c__Coriobacteriia	3.48307545	Ob_AN	3.072469712	1.66E-06
17	f__Atopobiaceae	3.478308604	Ob_AN	3.067325646	1.49E-06
18	p__Proteobacteria	5.612071479	Control	4.741389004	1.03E-06
19	c__Gammaproteobacteria	5.609327444	Control	4.73621017	1.94E-06
20	o__Burkholderiales	5.333327861	Control	4.51161631	0.001022
21	o__Pasteurellales	5.259306578	Control	4.493032101	0.000147
22	f__Pasteurellaceae	5.259306578	Control	4.493032101	0.000147
23	o__Cytophagales	0.598803401	Control	4.35014414	0.049276
24	g__Haemophilus	5.151185145	Control	4.336207654	0.007988
25	f__Burkholderiaceae	4.531748521	Control	4.197813437	3.12E-15
26	g__Lautropia	4.531748521	Control	4.197812719	3.18E-15
27	p__Fusobacteriota	4.994261683	Control	4.143072114	0.002268
28	c__Fusobacteriia	4.994261683	Control	4.143072114	0.002268
29	o__Fusobacteriales	4.994261683	Control	4.143072114	0.002268
30	g__Fusobacterium	4.905678264	Control	4.013564997	0.004639
31	f__Fusobacteriaceae	4.905678264	Control	4.013564954	0.004639
32	g__Aggregatibacter	4.423873762	Control	3.8543349	1.22E-06
33	g__Treponema	4.390442255	Control	3.85071428	1.12E-05
34	o__Spirochaetales	4.390810909	Control	3.841054126	1.13E-05
35	f__Spirochaetaceae	4.390810909	Control	3.841054126	1.13E-05
36	c__Spirochaetia	4.390810909	Control	3.841054126	1.13E-05

S/N	Biomarker	Logarithm value	Groups	LDA (Log10)	*p*-value
37	p__Spirochaetota	4.390810909	Control	3.841054009	1.13E-05
38	c__Clostridia	4.584415388	Control	3.694093287	0.044736
39	o__Flavobacteriales	4.491384407	Control	3.689725792	3.7E-05
40	g__Faucicola	0.850904028	Control	3.668624876	0.049276
41	g__Capnocytophaga	4.409875404	Control	3.64592808	6.8E-05
42	f__Flavobacteriaceae	4.409894103	Control	3.6454697	6.95E-05
43	g__Rubellimicrobium	1.161594197	Control	3.532020574	0.006185
44	o__Lachnospirales	4.274217657	Control	3.520727381	0.00694
45	g__Actinobacillus	3.992934479	Control	3.466638619	0.003591
46	f__Lachnospiraceae	4.241120435	Control	3.44902427	0.025287
47	g__Muribaculum	1.856353985	Control	3.444269166	0.049276
48	f__Clostridia_UCG_014	3.85237723	Control	3.326163097	3.17E-06
49	g__Clostridia_UCG_014	3.85237723	Control	3.326163097	3.17E-06
50	o__Clostridia_UCG_014	3.85237723	Control	3.326163097	3.17E-06
51	f__Muribaculaceae	3.565305249	Control	3.313542191	3.25E-05
52	g__Muribaculum	3.556732654	Control	3.305265799	3.25E-05
53	c__Gemmatimonadetes	2.555634648	Control	3.298550141	0.049276
54	g__Romboutsia	1.664207678	Control	3.287826783	0.002689
55	g__Olsenella	1.535294076	Control	3.2816242	0.029461
56	o__Gemmatimonadales	2.555634648	Control	3.187481822	0.049276
57	g__Catonella	3.800136668	Control	3.161813044	0.004517
58	f__Lentimicrobiaceae	3.567857713	Control	3.144767119	0.000662
59	g__Lentimicrobium	3.567857713	Control	3.144767119	0.000662
60	o__Sphingobacteriales	3.567857713	Control	3.144767119	0.000662
61	p__Gemmatimonadota	2.555634648	Control	3.10349625	0.049276
62	g__Pelospora	1.543223948	Control	3.101525343	0.005842
63	f__Tannerellaceae	3.538134551	Control	3.100135414	1.77E-06
64	o__Syntrophomonadales	1.543223948	Control	3.088851902	0.005842
65	f__Syntrophomonadaceae	1.543223948	Control	3.080458841	0.005842
66	g__Tannerella	3.505616551	Control	3.057495071	5.87E-06
67	g__Mycoplasma	3.451942134	Control	3.053023971	0.005389
68	f__Mycoplasmataceae	3.452341242	Control	3.052180786	0.0047
69	o__Mycoplasmatales	3.452341242	Control	3.052180786	0.0047
70	c__Alphaproteobacteria	3.409392101	Control	3.011514755	5.09E-06

Table S2 Details of Spearman correlation between the microbiota and predicted pathways related to Figure 6 and Figure S5.

Data1	Data2	*rho*	*p* value	Relation
Steroid hormone biosynthesis	uncultured_bacterium_g__Treponema	0.7460651	9.57221E-28	positive
Streptococcus_parasanguinis_g__Streptococcus	Toll and Imd signaling pathway	0.9027084	1.04407E-55	positive
Steroid hormone biosynthesis	Treponema_denticola_g__Treponema	0.8741282	5.89612E-48	positive
Eubacterium_sulci_g__[Eubacterium]_nodatum_group	Sesquiterpenoid and triterpenoid biosynthesis	0.7846487	2.49193E-32	positive
Steroid hormone biosynthesis	Treponema_sp._g__Treponema	0.7052526	1.01706E-23	positive
Basal transcription factors	uncultured_bacterium_g__Treponema	0.7542785	1.19301E-28	positive
Neuroactive ligand-receptor interaction	Prevotella_pleuritidis_g__Prevotella	0.9979533	1.8801E-177	positive
Glycosylphosphatidylinositol (GPI)-anchor biosynthesis	Treponema_denticola_g__Treponema	0.7723178	9.10533E-31	positive
Glycosylphosphatidylinositol (GPI)-anchor biosynthesis	Tannerella_forsythia_g__Tannerella	0.9993106	3.6168E-212	positive
Peptococcus_sp._g__Peptococcus	Synaptic vesicle cycle	0.8002058	1.86958E-34	positive
Peptococcus_sp._g__Peptococcus	Vasopressin-regulated water reabsorption	0.8002058	1.86958E-34	positive
Bacteroides_thetaiotaomicron_g__Bacteroides	Tetracycline biosynthesis	0.8894067	7.79245E-52	positive
Retrograde endocannabinoid signaling	uncultured_Clostridiales_g__Lachnospiraceae_NK4A136_group	0.7046371	1.15552E-23	positive
Prevotella_melaninogenica_g__Prevotella	Sphingolipid metabolism	0.8493089	0	positive
Prevotella_melaninogenica_g__Prevotella	Various types of N-glycan biosynthesis	0.7268493	0	positive
Fusobacterium_periodonticum_g__Fusobacterium	Stilbenoid, diarylheptanoid and gingerol biosynthesis	0.772486	8.68242E-31	positive
Toluene degradation	uncultured_bacterium_g__Lautropia	0.8636398	1.41698E-45	positive
Porphyromonas_endodontalis_g__Porphyromonas	Steroid hormone biosynthesis	0.7289409	5.74595E-26	positive
Rothia_mucilaginosa_g__Rothia	Sphingolipid signaling pathway	0.9190549	2.58578E-61	positive
Neisseria_oralis_g__Neisseria	Vibrio cholerae infection	0.7378612	7.08471E-27	positive

Data1	Data2	*rho*	*p* value	Relation
Steroid hormone biosynthesis	Treponema_medium_g__Treponema	0.8670253	2.54169E-46	positive
Apoptosis	Prevotella_melaninogenica_g__Prevotella	0.8018973	0	positive
Atrazine degradation	uncultured_bacterium_g__Lautropia	0.7988717	2.89212E-34	positive
Carbohydrate digestion and absorption	Prevotella_salivae_g__Prevotella	0.7424043	2.36044E-27	positive
Basal transcription factors	Treponema_medium_g__Treponema	0.7910619	3.4876E-33	positive
Atrazine degradation	Rothia_aeria_g__Rothia	0.7152675	1.21577E-24	positive
cAMP signaling pathway	Rothia_aeria_g__Rothia	0.9678024	5.8112E-90	positive
Lysosome	Prevotella_melaninogenica_g__Prevotella	0.7952621	0	positive
Other glycan degradation	Prevotella_melaninogenica_g__Prevotella	0.7579249	0	positive
Glycosaminoglycan degradation	Prevotella_melaninogenica_g__Prevotella	0.7181825	0	positive
Glycosphingolipid biosynthesis - ganglio series	Prevotella_melaninogenica_g__Prevotella	0.7271504	0	positive
Glycosphingolipid biosynthesis - globo and isoglobo series	Prevotella_melaninogenica_g__Prevotella	0.8012552	0	positive
D-Arginine and D-ornithine metabolism	Fusobacterium_periodonticum_g__Fusobacterium	0.7252507	1.33336E-25	positive
Flavonoid biosynthesis	Fusobacterium_periodonticum_g__Fusobacterium	0.772486	8.68242E-31	positive
Fluorobenzoate degradation	uncultured_bacterium_g__Lautropia	0.8788768	4.18303E-49	positive
Prevotella_melaninogenica_g__Prevotella	Protein digestion and absorption	0.7353601	0	positive
Pancreatic secretion	Prevotella_salivae_g__Prevotella	0.7460216	9.67621E-28	positive
Prevotella_salivae_g__Prevotella	Salivary secretion	0.7460216	9.67621E-28	positive
Polycyclic aromatic hydrocarbon degradation	Rothia_aeria_g__Rothia	0.8126692	2.68736E-36	positive
Retrograde endocannabinoid signaling	uncultured_bacterium_g__Mucispirillum	0.9109928	2.05456E-58	positive

Data1	Data2	*rho*	*p* value	Relation
Clostridiales_bacterium_g__Clostridia_vadinBB60_group	Steroid hormone biosynthesis	0.7257303	1.1961E-25	positive
Bile secretion	uncultured_bacterium_g__Cardiobacterium	0.876064	2.0316E-48	positive
Cell cycle	Mogibacterium_pumilum_g__Mogibacterium	0.9256457	6.42646E-64	positive
Cell cycle - yeast	Mogibacterium_pumilum_g__Mogibacterium	0.9252552	9.3084E-64	positive
Steroid biosynthesis	uncultured_bacterium_g__Succinivibrio	0.7050659	1.05725E-23	positive
Bacteroides_stercoris_g__Bacteroides	Glycosphingolipid biosynthesis - lacto and neolacto series	0.9452326	2.30379E-73	positive
